# Extra-long interglacial in Northern Hemisphere during MISs 15-13 arising from limited extent of Arctic ice sheets in glacial MIS 14

**DOI:** 10.1038/srep12103

**Published:** 2015-07-10

**Authors:** Qingzhen Hao, Luo Wang, Frank Oldfield, Zhengtang Guo

**Affiliations:** 1 Key Laboratory of Cenozoic Geology and Environment, Institute of Geology and Geophysics, Chinese Academy of Sciences, P.O. Box 9825, Beijing 100029, China; 2 School of Environmental Sciences, University of Liverpool, Liverpool L69 7ZT, UK

## Abstract

Knowledge of the behavior of Northern Hemisphere (NH) ice sheets over the past million years is crucial for understanding the role of orbitally driven insolation changes on glacial/interglacial cycles. Here, based on the demonstrable link between changes in Chinese loess grain-size and NH ice-sheet extent, we use loess grain-size records to confirm that northern ice-sheets were restricted during marine oxygen isotope stage (MIS) 14. Thus, an unusually long NH interglacial climate of over 100 kyr persisted during MISs 15−13, much longer than expected from marine oxygen isotope records. Taking a global view of the paleoclimate records, MIS 14 inception seems to be a response to changes in Antarctic ice-sheets rather than to NH cooling. Orbital configuration in the two Polar regions shows that the onset of MIS 14 was forced by austral insolation changes, rather than by boreal summer insolation, as Milankovitch theory proposes. Our analysis of MIS 14 raises the possibility that southern insolation forcing may have played an important role in the inception of several other glacials. We suggest that the extra-long NH interglacial climate during MISs 15−13 provided favorable conditions for the second major dispersal episode of African hominins into Eurasia.

The marine δ^18^O records of benthic foraminifera show that the growth and reduction of global ice volume exhibited dominant quasi-periods of 100 kyr since the Mid-Pleistocene transitions of 1200−800 kyr ago^1^,^2^. Normal interglacials generally span two or three precession cycles with a maximum durations of 60 kyr. The temperature record of the last 800 kyr from the EPICA ice core^3^ shows a strikingly consistent sequence. However, some records, e.g. biogenic carbonate^4^ and ice-rafted-debris (IRD) content^5^ in North Atlantic sediments, suggest that MIS 14 was characterized by much less severe glacial conditions, implying that high northern latitudes experienced an extra-long predominantly interglacial style climate during MISs 15–13. Obtaining conclusive evidence for this is hampered by the lack of direct sedimentary records of ice sheets older than the last interglacial in Arctic regions. Moreover, the benthic δ^18^O records represent the integrated effect of the ice-volume signals from the both hemispheres^6^. Confirming the extent of Northern Hemisphere ice sheets during MIS 14 can provide new insight into the 100-kyr climate cycles and improve our understanding of the forcing mechanism of the Pleistocene glacial and interglacial cycles.

In the MIS sequence, MIS 14 stands out as a short and mild glacial epoch in many records^7^ spanning the last 0.7 Myr, throughout which the climate was dominated by 100-kyr cycles. During MIS 14, the climate exhibited a complicated and unusual global configuration. The paleoclimate records from Antarctic ice cores and from deep sea sediment records in middle- and high- latitudes of the Southern Hemisphere indicate relative severe glacial conditions^3^,^8^. However, records from the Northern Hemisphere suggest that MIS 14 was a much warmer glacial period than other glacial epochs in the last 800 kyr^4^,^7^,^9^. Therefore, there seems to be a strong inter-hemispheric asymmetry in MIS 14. Neither the buildup and extent of ice sheets in high northern latitudes, nor the mechanisms responsible for the imbalance between the records from each hemisphere are clearly understood.

The link between the physical properties of Chinese loess and the extent of Arctic ice sheets has provided new insight into climate changes at high northern latitudes^10^. The spatially coherent Quaternary loess-paleosol sequences in the Chinese Loess Plateau (CLP) have long been regarded as near-continuous records of Asian monsoon climate. Variability in the grain-size of Chinese loess deposits, mainly carried by the East Asian winter monsoon (EAWM), tracked Arctic climate variability because the strength of the EAWM is tightly linked via the Siberian High anticyclone to variations in Northern Hemisphere ice sheets^11^. The close coupling between high northern latitude cooling and increased dust activity in Asian Interior deserts can be confirmed on timescales ranging from decadal to orbital as summarized in Hao *et al*.^10^. The Chinese loess records can thus provide independent evidence for the changes in Northern Hemisphere ice volume as grain-size variability is closely linked with the large-scale growth of ice sheets.

Evidence from two loess-paleosol sections, Yimaguan (YMG) and Luochuan (LC)^10^ 160 km apart, is presented here. These two sections provide parallel loess records that represent the orbital scale changes in East Asian monsoon climate over the CLP region (Supplementary Fig. 1 and 2). The middle and upper parts of the two sections, spanning units L9 to S0 and covering the last 900 kyr, were sampled. The samples were usually analyzed at 5-cm intervals for both sections, with 2.5-cm intervals for paleosol S5. The 5-cm-interval represents an average time resolution for loess and paleosol layers of 0.36 and 0.89 kyr for YMG, 0.56 and 1.24 kyr for LC, respectively. The 2.5-cm-interval, represents 0.56 kyr and 0.83 kyr for S5 in YMG and LC sections, respectively. The chronology of the two sections was generated by correlation of the studied sequences with the benthic δ^18^O stack LR04^12^, outlined in Hao *et al*.^10^.

Frequency dependent magnetic susceptibility, χ_fd_, and the grain-size fraction >32 μm (GT32) are used as the proxies for East Asian summer monsoon (EASM)^13^,^14^ and EAWM^15^,^16^, respectively. Measurements of grain size and magnetic susceptibility were made using the routine methods described in Hao *et al*.^10^ which also outlines the justification for the use of the two proxies.

## Results

**Figure 1:**
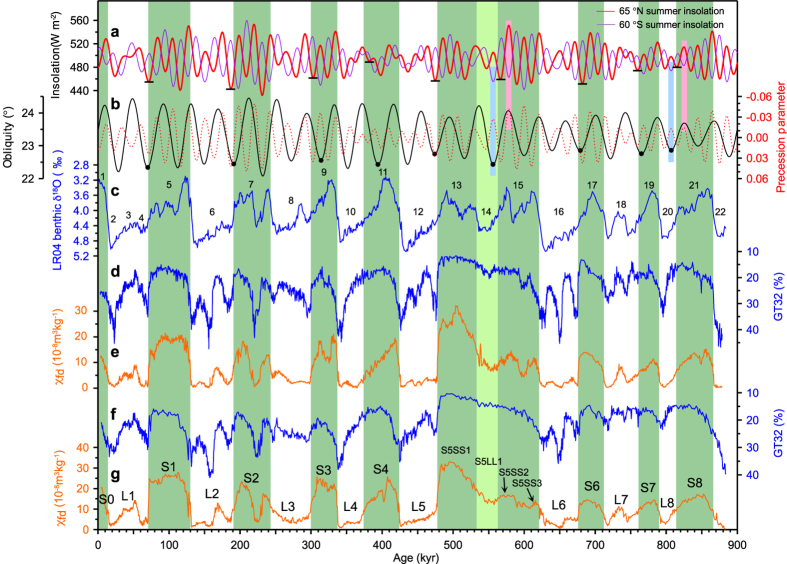
Comparison of the East Asian winter monsoon (EAWM) and summer monsoon (EASM) proxy records in loess with marine and Earth’s orbital records of the last 880 kyr. **a**, 21 June insolation 65° N and 21 December insolation 60°S^59^. Short black horizontal bars mark the insolation minima at 65°N coincident with each glacial inception. **b**, Earth’s obliquity, and precession parameters^59^ on a reversed scale. **c**, Benthic δ^18^O stack LR04^12^. Numbers indicate Marine Isotopic Stages. **d** and **e**, Grain-size data expressed as GT32 (>32 μm particle content), and frequency-dependent magnetic susceptibility (χ_fd_) in the Yimaguan loess section, respectively. **f** and **g**, GT32 and χ_fd_ data in the Luochuan loess section, respectively. The major loess and paleosol units are labeled in **g**. The green shaded bars indicate interglacial MIS stratigraphy, and the light green one highlights the “glacial” MIS 14. The pink bars in **a** and **b** indicate late interglacial periods with close alignment of obliquity and boreal precessional insolation maxima, and light blue ones, early glacial periods with close alignment of obliquity and austral precessional insolation minima. The solid circles on the obliquity curve in **b** indicate the obliquity minima around the glacial inceptions used in the calculation in the Fig. 3. Note the obliquity minima at 314 kyr and 394 kyr are much earlier than the inceptions of MISs 8 and 10.

The GT32 and χ_fd_ proxy records from the YMG and LC sections exhibit spatially coherent changes (Fig. 1d–g). Glacial loess is generally characterized by coarse grained sediments, hence higher GT32 values and low χ_fd_ values, indicating overall strong EAWM winds and arid conditions. The interglacial soils are characterized by low GT32 and high χ_fd_ values, indicating weak EAWM winds and humid conditions. The interpretation of these well-established properties has been reinforced by parallel changes in a range of physical, geochemical and biological proxies^17^.

According to the degree of coupling between grain-size and χ_fd_ records (Fig. 1d–g), two types of interglacial-glacial transitions have been recognized and their incidence explained^10^. The most frequent is characterized by a rapid grain-size increase at the contact between each loess layer and the underlying soil (S1-L1, S2-L2, S3-L3, S5-L5, S6-L6). Less frequently, there is a delayed increase in grain-size above the contact (S4, S7, S8). In the latter cases, there is a delayed buildup of Arctic ice-sheets by up to 20 kyr during the periods of low eccentricity and precessional variability at ~400 kyr intervals^10^. The MIS 14 loess provides the only exception to the above two types of change. It is characterized by the following features: 1). The grain-size records show a weak increase with a much reduced amplitude at the inception of MIS 14. This is in sharp contrast to the amplitude of change recorded in MIS 4 (lower L1), MIS 6 (L2), MIS 8 (L3), MIS 12 (L5) and MIS 16 (L6); 2). Fine-grained loess deposition persisted throughout MIS 14 without any interval of coarse-grained dust accumulation such as occurred during other glacial periods (Fig. 1). The above features can be seen in all the median grain-size or mean grain-size records from loess sections throughout the CLP (Supplementary Fig. 2). In the western CLP, the MIS 14 deposits are comparable to the stadial dust deposits within the major interglacial soil units, e.g., S1 (MIS 5) and S3 (MIS 9) (Supplementary Fig. 2a,2b).

Rapid coarsening of loess is closely linked with the rapid growth of ice-sheets in high northern latitudes^10^,^11^. Therefore the persistence of fine-grained loess deposition throughout MIS 14 on the CLP (Fig. 1 and Supplementary Fig. 2) indicates that the extent of continental ice sheets on the high northern latitudes remained limited during MIS 14.

## Discussion

**Figure 2:**
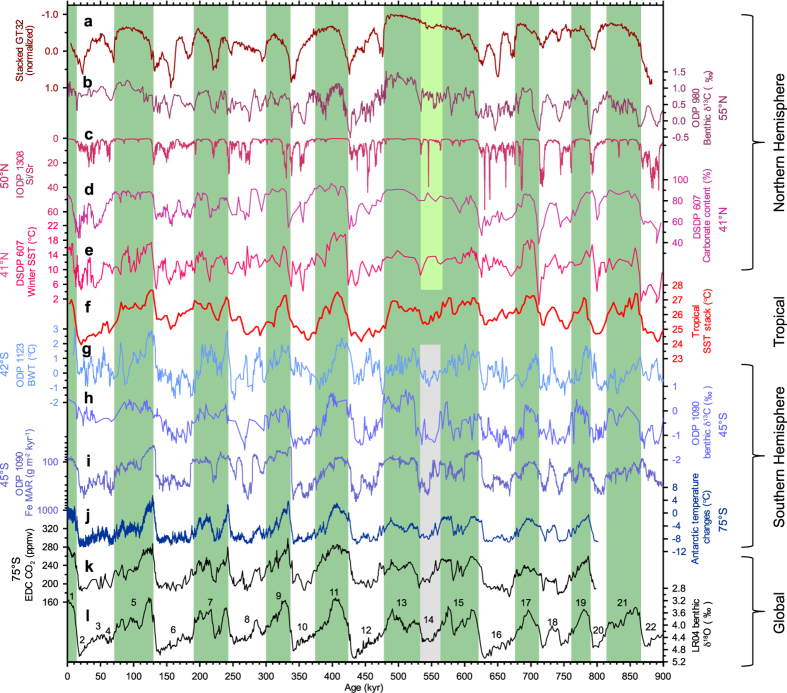
Comparison of the stacked loess records of the East Asian winter monsoon with the global paleoclimate records. **a−e**, Northern Hemisphere records. **a**, stacked grain-size GT32 record of East Asian winter monsoon. **b**, Benthic δ^13^C records from ODP 980^18^,^19^. **c**, Si/Sr ratio, reflecting layers that are poor in biogenic carbonate and relatively rich in detrital silicate minerals at IODP U1308 within the IRD belt^5^. **d** and **e**, Carbonate content and surface sea temperature (SST) in winter season from DSDP 607, respectively^4^. **f**, Tropical SST stack^34^. **g−j**, Southern Hemisphere records. **g**, Bottom water temperature (BWT) record from Mg/Ca of ODP 1123^8^. **h** and **i**, Benthic δ^13^C^23^ and mass accumulation rate (MAR) of eolian iron flux^24^ from ODP 1090, respectively. **j**, Air temperature changes from EPICA ice core^3^. **k**, Antarctic CO_2_ concentation record^60^. **l**, Benthic δ^18^O stack LR04^12^. All the records from ocean deposits were calibrated to the timescale of LR04^12^, except the Fe MAR records of ODP 1090 in **l** which were tuned according to the EPICA EDC3 time scale^24^. Data in **b** and **g** are smoothed with a three-point running mean. The green shaded bars indicate interglacial MIS stratigraphy, and the light green and light gray ones highlight inter-hemispheric asymmetry during the “glacial” MIS 14.

The small extent of Northern Hemisphere ice sheets during MIS 14 indicated by our study is consistent with the records from northern Atlantic and Eurasian continent. The records from the northern Atlantic, spanning 41°–61° N indicate that compared with other glacial periods, MIS 14 is characterized by relatively enriched benthic δ^13^C^18^,^19^ (Fig. 2b), less severe ice-rafting activity^5^,^20^,^21^ (Fig. 2c), a higher production of biogenic carbonate and warmer winter seasons^4^ (Fig. 2d,e, and Supplementary Fig. 3b–f and Information). The long time-series of U^k’^_37_ –based SST reconstructions at ODP 982 by Lawrence *et al*.^20^ shows that at latitudes >50°N, high amplitude variations similar to those typical of the Pleistocene began as early as 4 Ma. The reconstructed variations in SST cannot therefore be simply a function of glacial/interglacial alternations. Doubt is also cast on their reliability in this regard by the sharp contrast between the SST record for MIS 14, with typical glacial values, and the magnetic susceptibility record from the site, showing, exceptionally, the absence of IRD signals^20^. The warm climate conditions during MIS 14 are also indicated by an exceptional absence of mountain glaciers in the lake Baikal region^22^ and stadial-like arboreal vegetation in NE Greece^9^ (Supplementary Fig. 3g,3h).

Many records from mid-high latitudes in the Southern Hemisphere, by contrast, indicate cold climate conditions. These lines of evidence include cold bottom water in the southwest Pacific Ocean^8^ (Fig. 2g), depleted benthic δ^13^C^23^ and high dust flux^24^ in the South Atlantic sector of the Southern Ocean (Fig. 2h,i), and low temperatures in the Antarctic region^3^ (Fig. 2j), which differ relatively little from those prevailing during other glacial periods. These records indicate broadly similar glacial condition for MIS 14. It follows that during MIS 14 there is a marked inter-hemispheric asymmetry. The present evidence suggests that the climate in high southern latitudes may be the primary factor driving global climate towards the special configuration of glacial conditions during MIS 14. This is in strong contrast to the traditional view that throughout the Pleistocene, feedback from expanding Northern Hemisphere ice-sheets was the dominant trigger for global glacial climates.

The overall mild NH climate in MIS 14, with strong hemispheric asymmetry, is linked with both external forcing and internal processes within the Earth climate system. External forcing here refers to the changes in solar insolation at high latitudes caused by the Earth’s orbit. Internal processes include interactions (responses and feedbacks) among the elements within the Earth climate system. Here we focus on three widely proposed mechanisms: snow-ice feedback, the thermohaline circulation and the influence of tropical sea surface temperature (SST)^25^.

The Milankovitch hypothesis proposes that changes in boreal summer insolation control the Northern Hemisphere ice sheets through their influence on the ablation of snow and ice^26^. Climate modeling studies show that the reduction in boreal summer insolation is a prerequisite for glacial inception^27^,^28^, and a low obliquity value plays an important role in determining the strength of the inception processes through acting to delay the spring melt season at higher latitudes and thus extend winter snow accumulation^27^. Therefore, the close alignment of obliquity and Northern Hempispere precessional insolation minima are most favorable for transitions from interglacial to glacial climate condition, e.g., as seen in onset of MIS 4 and MIS 6 (Fig. 1a−c).

**Figure 3:**
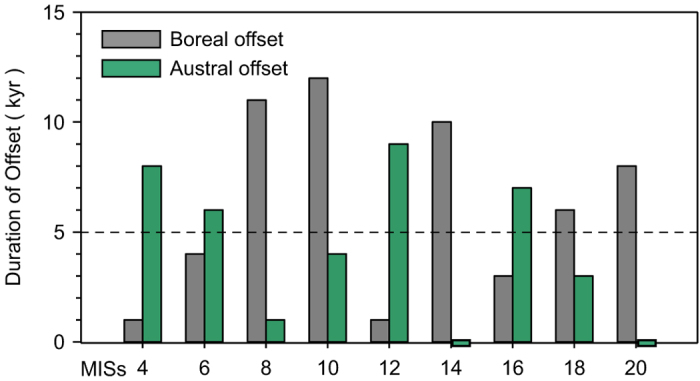
Alignment of the Earth’s obliquity and the boreal and austral precessional insolation minima around glacial inceptions over the last 900 kyr. The alignment is expressed by the duration of offset between the insolation minimum and the nearest obliquity minimum as shown in Fig. 1b. The horizontal dashed line shows the empirical value of 5 ka, below which the alignment of obliquity and precessional insolation minima are considered to be in phase^3^.

The orbital configuration in the period from late MIS 15 to MIS 14 is unique in the context of interglacial-glacial transitions over the last 900 kyr. First, the offset in the timing of obliquity and precessional insolation minima, up to 10 kyr, is one of the largest in glacial inceptions (MISs 4, 6, 8, 12, 14 and 16) in the last 900 kyr (Fig. 1a,b and 3). In the early part (563−549 kyr BP) of MIS 14 (563−533 kyr BP) (Fig. 1a,b), this configuration led to changes in precession-linked insolation that were exactly in anti-phase with the obliquity-linked changes. Under these configurations, the conditions for preventing spring snow melting and summer snow melting are not in phase. Secondly, preceding the MIS 14 inception, the precessional insolation maximum at 578 kyr is in phase with the orbital obliquity maximum (Fig. 1a,b). This kind of configuration is thought to be responsible for the deglaciation^29^,^30^,^31^ and strong interglacial climate during early stage of MISs 5, 9, 11 and 19^3^. However, during the late stages of interglacials, this kind of configuration only occurred in MISs 15 and 21. Furthermore, the insolation maximum at 578 kyr is one of the three highest maxima (the other two are at 127 kyr and 220 kyr, respectively) (Fig. 1a). The extreme warmth during MIS 15a has been identified by simulated surface temperatures averaged by hemispheres^32^. Therefore, the insolation forcing preceding the MIS 14 inception would have caused the melting of the ice and snow that had accumulated during the previous stadial within MIS 15 to a much lower level than was the case during other interglacials. As a result, the orbital configuration prior to the MIS 14 and within MIS 14 constitutes the most unfavorable condition for the development of a severe boreal glacial climate over the last 900 kyr.

Internal forcing within the climate system^28^,^33^ also played an important role in the mild climate of MIS 14. The first mechanism is snow- and ice- albedo feedback. The late-middle Pleistocene glacial-interglacial alternations are generally characterized by rapid deglaciation, followed by a gradual return to full glacial conditions. The gradual accumulation of ice towards the end of each interglacial is thought to be an important boundary condition for glacial inception^30^. Climate modeling studies shows that at the initial stage of glacial inception, strong snow albedo feedback played a major role in driving the climate transition from interglacial to glacial states^28^. As stated above, the strong ablation of ice in late MIS 15, induced by synchronous precessional summer insolation and obliquity maxima, led to the existence of much lower volumes of ice. Thus, MIS 14 would have started with a much reduced ice-snow cover in high northern latitudes, unfavorable for the transition from an interglacial to a glacial state. The relative high CO_2_ concentrations compared to other glacial inceptions (Fig. 2k) also limited the degree of internal forcing towards colder conditions.

The second mechanism is linked to the intensity of the thermohaline circulation. The benthic δ^13^C records in North Atlantic indicate a relatively high level of productivity in North Atlantic Deep Water throughout of MIS 14^18^,^19^ (Fig. 2b), which would draw more warm surface seawater from the low-latitude ocean into the sub-polar and polar North Atlantic.

The third mechanism involves tropical sea surface temperatures. The tropical oceans have a relative high SSTs during MIS 14 compared with many other Pleistocene glacials^34^ (Fig. 2f). In the Atlantic sector, the moderate intensity of the thermohaline circulation, indicated by benthic δ^13^C records^4^,^18^,^19^ (Fig. 2b), would also have transported more heat from the tropical ocean to high northern latitudes. In the Pacific sector, the relative high SST in the eastern equatorial Pacific^34^ may also be unfavorable to ice sheet growth^25^.

The combined influence of orbitally generated external boundary conditions and internal forcing led to much warmer conditions at high northern latitudes during MIS 14 than during typical glacials. The mild-warm climate in Northern Hemisphere appears to have extended further south to low southern latitudes. This is strongly supported by diatom evidence from the South Atlantic (~30 °S), indicating the frequent influence of warm sea-water originating in the low-latitude Indian Ocean during MIS 14^35^ (Supplementary Fig. 3i).

In contrast to high northern latitudes, the high southern latitudes experienced an orbital configuration favoring the accumulation of snow and ice in Antarctica. The low austral insolation minima coinciding with low obliquity minima at 556 kyr ago (Fig. 1a,b), and the synchronous decline in austral summer insolation and obliquity between 568 and 556 kyr ago may have been the primary factors driving the Antarctic climate into typical glacial conditions. Glacial conditions could be intensified by sea-ice feedback triggered by austral insolation changes^36^,^37^,^38^. The southern ocean sea-ice during MIS 14 reached an extent similar to that during other typical glacial periods^39^. Typical glacial conditions have also been inferred from modeling results^40^. The buildup of Antarctic ice sheets during MIS 14 affected the formation of bottom water sourced from the dense sinking water around Antarctica, which subsequently led to low benthic δ^13^C values typical of glacial conditions in the South Atlantic sector of the Southern Ocean^23^ (Fig. 2h), a partial decrease in benthic δ^13^C values in the North Atlantic^4^,^41^, and low southern Pacific bottom water temperatures^8^ (Fig. 2g).

In summary, grain-size records in Chinese loess confirm an exceptionally limited extent of the Northern Hemisphere ice sheets during MIS 14. It seems that the MIS 14 climate in the Northern Hemisphere was more comparable to that prevailing during mild stadials within intergalcials, suggesting that the Northern Hemisphere experienced a super-interglacial style climate in terms of duration during MISs 15−13, 621−478 kyr ago. Taking a global view of the paleoclimate records, the MIS 14 climate demonstrates a strong inter-hemispheric asymmetry, with a warmer Northern Hemisphere and colder high southern latitudes. Present evidence shows that MIS 14 is a period in the late-middle Pleistocene when the austral summer insolation triggered an atypical glacial epoch.

It has long been accepted that the glacial-interglacial cycles were synchronous in the Northern and Southern Hemispheres, triggered by summer insolation at high northern latitudes as proposed by Milankovitch^26^. However, the key physical mechanisms are far from well understood^30^,^42^. To understand the relationship of insolation and the glacial cycles, numerous investigations have focused on the orbital configuration or the insolation around glacial terminations^31^,^36^,^43^,^44^,^45^, because the magnitude and abruptness of changes at the terminations facilitate accurate identification of climate transitions. In contrast, the relationship between glacial inception and regional insolation forcing is less clear due to the gradual changes in the marine δ^18^O records at the interglacial-to-glacial transitions, as, for example, during marine oxygen isotope stage (MIS) 11/10 and MIS 9/8, although the timing of the two recent transitions at MIS 7/6 and 5/4 appears to be consistent with Northern Hemisphere forcing^29^,^30^. Deciphering MIS 14 with strong hemispheric asymmetry decreases the uncertainty in the correlation of orbital forcing and gradual climate changes during glacial inception, and provides a convincing pointer to the pattern of insolation responsible for glacial inception.

Our analysis of MIS 14 raises the possibility that over the last 900 kyr, southern insolation forcing may have played an important role in the inception of several other glacials. The long-term variations in insolation mainly depend on the alignment of precession and obliquity^46^. Here, we use the empirical threshold value of 5 kyr^3^ to identify instances when the alignment of obliquity and precessional insolation minima are considered to be in phase. Orbital alignment with an offset in the timing of obliquity and precessional insolation minima of less than 5 kyr favors the local buildup of ice sheets. During the last nine glacial-interglacial cycles, the inceptions of MISs 8, 10, 18 and 20 are, like MIS 14, characterized by an offset of less than 5 kyr in the Antarctic region and a long duration of offset in the Arctic region (Fig. 3). For the cases of MISs 10, 18 and 20, the orbital configurations support our interpretations that these three glacials began with early build-up of ice volume in the Antarctic region^10^. During the transition from MIS 11 to 10, the southern insolation forcing may have been strengthened by the prolonged overall decrease in insolation from 418 kyr to 390 kyr, spanning one and a half precessional cycles (Fig. 1a). As for MIS 8, the orbital configuration in the Antarctic region may have been partly responsible for the transition from interglacial to glacial conditions. The geological evidence and orbital configuration therefore raise the possibility that over the past million years, several glacial periods pre-MIS 11, such as MISs 14, 18 and 20, may have been triggered by Southern Hemisphere insolation forcing.

The extra-long and warm, predominantly interglacial style climate in the Northern Hemisphere during MISs 15−13 may have had a profound influence on the migration of early humans within the context of alternating glacial and interglacial climates. The role of climate in three major dispersal episodes^47^,^48^ has long been a highly contentious issue. The Earth had begun to experience more severe glacial climate from 1.2 Ma onwards, especially after 0.7 Ma when global ice volume started to be dominated by 100-kyr cycles. Before MIS 15, northwestern Eurasia experienced at least two severe glaciations during MISs 24–22 and MIS 16, respectively^49^,^50^. The cold glacial climate and consequent deterioration in ecosystems had led to southward retreat, even regional extinction of the hominins^51^,^52^,^53^. It is argued that hominins abandoned Eurasia between 40−50° N for up to 80% of the last 500−600 kyr^51^. However, genetic analysis reveals a major expansion of African hominins about 600 kyr ago, the second one of the three episodes of “out of Africa”^47^,^48^. This is in accordance with the archaeological evidence showing synchronous occurrence of similar forms of hominins on both sides of the Mediterranean and the emergence of Acheulean bifacial handaxes of African origin in Eurasia, even as far north as 50~53 °N, ~500−600 kyr ago^54^,^55^,^56^. Strikingly, the Paleolithic tools of MIS 14 age have also been found in Tajikistan, central Asia^51^, even in northern France^57^,^58^, in sharp contrast to other, typical, mid-Pleistocene glacials which are generally without evidence of hominin occupation in these regions. This implies that during MIS 14 the regions between 40−50° N had been warm enough to maintain the hominin occupation. This may have provided an opportunity for the early hominins to improve their adaptability to increasing seasonal contrasts and cold winters in the northern regions. On the other hand, these regions may have served as much more northerly refugia for hominins during MIS 14, compared with those in other glacials, facilitating hominin expansion further northward in the subsequent interglacial. Therefore, we propose that the extra-long duration of interglacial/mild stadial climates during MIS 15−13 may have provided favorable conditions over 100 kyr for the dispersal of African hominins into the Eurasia region during the middle Pleistocene, leading to sustainable occupation.

## Additional Information

**How to cite this article**: Hao, Q. *et al*. Extra-long interglacial in Northern Hemisphere during MISs 15-13 arising from limited extent of Arctic ice sheets in glacial MIS 14. *Sci. Rep*. **5**, 12103; doi: 10.1038/srep12103 (2015).

## Supplementary Material

Supplementary Information
