# Supplementary material for: Extra-long interglacial in Northern Hemisphere during MISs 15-13 arising from limited extent of Arctic ice sheets in glacial MIS 14
Source: Sci Rep. 2015 Jul 10;5:12103. doi: 10.1038/srep12103 (PMC4498323; doi:10.1038/srep12103)
Supplement: Supplementary Information [file srep12103-s1.pdf]

Supplementary Information

Extra-long interglacial in Northern Hemisphere during MISs 15-13 arising from limited extent of Arctic ice sheets in glacial MIS 14

Qingzhen Hao[1,*], Luo Wang[1,*], Frank Oldfield[2], Zhengtang Guo[1]

[1]Key Laboratory of Cenozoic Geology and Environment, Institute of Geology and Geophysics, Chinese Academy of sciences, P.O. Box 9825, Beijing 100029, China.

[2]School of Environmental Sciences, University of Liverpool, Liverpool L69 7ZT, UK.

* Corresponding author. Qingzhen Hao, E-mail: haoqz@mail.iggcas.ac.cn; Luo Wang, E-mail: wangluo@mail.iggcas.ac.cn.

Consistency in the grain size records across the Chinese Loess Plateau

Nine published loess-paleosol sequences with grain-size records, mostly spanning the last 900 kyr have been compiled[1-5] (Fig. S1b and S2). These sections span most of the Chinese Loess Plateau (CLP) from 34.43° N to 40.50° N and 102.82° E to 115.17° E (Fig. S1b), and confirm the widespread consistency of orbital-scale changes in grain-size, in terms of the relative amplitude of grain-size variations (Fig. S2). In the western CLP, the MIS 14 deposits are comparable to the stadial dust deposits within the major interglacial soil units, e.g., S1 and S3 (Fig. S2a, S2b).

Additional Evidence from the Atlantic and Eurasian continent

Additional evidence from the North Atlantic, low-latitudes of South Atlantic and the Eurasian continent was compiled (Fig. S3). The deep-sea records from the North Atlantic cover the vast region 41°–61° N. The IRD records[7,8] (Fig. S3b, S3c), north of the IRD Belt[9], confirm the less severe ice-rafting activity during MIS 14 as do those in IRD belt itself[10,11](Fig. 2c and Fig. S3e). The bulk $\delta^{18}O$ reflects the proportion of detrital to biogenic carbonate, because the $\delta^{18}O$ of detrital carbonate is ~9‰ lower than foraminifer carbonate[12]. IODP U1308 shows the high bulk $\delta^{18}O$ values during MIS 14, typical of interglacial values, indicating high foraminifer production[12] (Fig. S3d). Carbonate percentage in the Atlantic mainly reflects production of planktonic organisms in pelagic sediments. The low-resolution $CaCO_3$ record at Site 607[13] is confirmed by high-resolution records at Site U1313[11] (Fig. S3f).

On the Eurasian continent, the terrestrial records also confirm the persistence of warm conditions during MIS 14. The glacial sediments in Baikal generally contain IRD produced by mountain glaciers descending into the lake. The IRD sediments have high magnetic susceptibility. The particularly low susceptibility values, reflecting a lack of glacial IRD signals, indicate there were no mountain glaciers during MIS 14[14] (Fig. S3g). The long-term pollen records from NE Greece provide an excellent record

of the vegetation's response to the glacial-intergalcial climate cycles[15]. The record shows that during MIS 14 arboreal vegetation persisted throughout, with values, at their minimum, either comparable to those during stadial stages within the interglacial periods, e.g., MISs 5 and 9, or higher as in the case of the stadial within MIS 15.

During MIS14, an almost monospecific ooze of the giant diatom *Ethmodiscus* rex, up to 1.5 m thick, was deposited in the subtropical South Atlantic[16-18]. This diatomaceous ooze indicates the frequent influence of warm, silicate-rich water borne by the Agulhas current originating in the low-latitude Indian Ocean, which provides the soluble Si to enable the growth of *Ethmodiscus*[18]. The sea surface temperatures (SST) reconstructed from the Mg/Ca ratio in planktonic *Globigerinoides ruber* in core GeoB 3801-6 support this interpretation[18] (Fig. S3i), confirming the influence of warm Northern Hemisphere and tropical waters in the subtropical South Atlantic within the context of strong hemispheric asymmetry.

References

1. Wu, G., Pan, B., Guan, Q. & Xia, D. Terminations and their correlation with solar insolation in the Northern Hemisphere: a record from a loess section in Northwest China. *Palaeogeogr Palaeoclimatol Palaeoecol* **216**, 267-277 (2005).
2. Sun, Y. *et al.* East Asian monsoon variability over the last seven glacial cycles recorded by a loess sequence from the northwestern Chinese Loess Plateau. *Geochem Geophy Geosy* **7**, Q12Q02, doi 10.1029/2006GC001287 (2006).
3. Xiong, S. F., Ding, Z. L. & Liu, T. S. Climatic implications of loess deposits from the Beijing region. *J Quat Sci* **16**, 575-582 (2001).
4. Ding, Z. L. *et al.* Stacked 2.6-Ma grain size record from the Chinese loess based on five sections and correlation with the deep-sea $\delta^{18}O$ record. *Paleoceanography* **17**, doi 10.1029/2001PA000725 (2002).
5. Sun, Y., Clemens, S. C., An, Z. & Yu, Z. Astronomical timescale and palaeoclimatic implication of stacked 3.6-Myr monsoon records from the Chinese

Loess Plateau. *Quat Sci Rev* **25**, 33-48 (2006).

6. Hao, Q. Z. *et al.* Delayed build-up of Arctic ice sheets during 400,000-year minima in insolation variability. *Nature* **490**, 393–396 (2012).
7. Wright, A. K. & Flower, B. P. Surface and deep ocean circulation in the subpolar North Atlantic during the mid-Pleistocene revolution. *Paleoceanography* **17**, 1068, doi 10.1029/2002PA000782 (2002).
8. McManus, J. F., Oppo, D. W. & Cullen, J. L. A 0.5-million-year record of millennial-scale climate variability in the North Atlantic. *Science* **283**, 971-975 (1999).
9. Ruddiman, W. F. North-Atlantic Ice-Rafting - Major Change at 75,000 Years before Present. *Science* **196**, 1208-1211 (1977).
10. Hodell, D. A., Channell, J. E. T., Curtis, J. H., Romero, O. E. & Rohl, U. Onset of "Hudson Strait" Heinrich events in the eastern North Atlantic at the end of the middle Pleistocene transition (similar to 640 ka)? *Paleoceanography* **23**, PA4218, doi 10.1029/2008PA001591 (2008).
11. Stein, R., Hefter, J., Grutzner, J., Voelker, A. & Naafs, B. D. A. Variability of surface water characteristics and Heinrich-like events in the Pleistocene midlatitude North Atlantic Ocean: Biomarker and XRD records from IODP Site U1313 (MIS 16-9). *Paleoceanography* **24**, PA2203, doi 10.1029/2008PA001639 (2009).
12. Hodell, D. A. & Curtis, J. H. Oxygen and carbon isotopes of detrital carbonate in North Atlantic Heinrich Events. *Marine Geology* **256**, 30-35 (2008).
13. Ruddiman, W., Raymo, M., Martinson, D., Clement, B. & Backman, J. Pleistocene evolution: Northern Hemisphere ice sheets and North Atlantic Ocean. *Paleoceanography* **4**, 353-412 (1989).
14. Prokopenko, A. A. *et al.* Muted climate variations in continental Siberia during the mid-Pleistocene epoch. *Nature* **418**, 65-68 (2002).
15. Tzedakis, P. C., Hooghiemstra, H. & Palike, H. The last 1.35 million years at

Tenaghi Philippon: revised chronostratigraphy and long-term vegetation trends. *Quat Sci Rev* **25**, 3416-3430 (2006).

16. Romero, O. & Schmieder, F. Occurrence of thick Ethmodiscus oozes associated with a terminal Mid-Pleistocene Transition event in the oligotrophic subtropical South Atlantic. *Palaeogeogr Palaeocl* **235**, 321-329 (2006).
17. Schmieder, F., von Dobeneck, T. & Bleil, U. The Mid-Pleistocene climate transition as documented in the deep South Atlantic Ocean: Initiation, interim state and terminal event. *Earth Planet Sc Lett* **179**, 539-549 (2000).
18. Rackebrandt, N., Kuhnert, H., Groeneveld, J. & Bickert, T. Persisting maximum Agulhas leakage during MIS 14 indicated by massive Ethmodiscus oozes in the subtropical South Atlantic. *Paleoceanography* **26**, PA3202, doi 10.1029/2010PA001990 (2011).
19. Lisiecki, L. & Raymo, M. A Pliocene-Pleistocene stack of 57 globally distributed benthic δ18O records. *Paleoceanography* **20**, PA1003. 10.1029/2004PA001071 (2005).

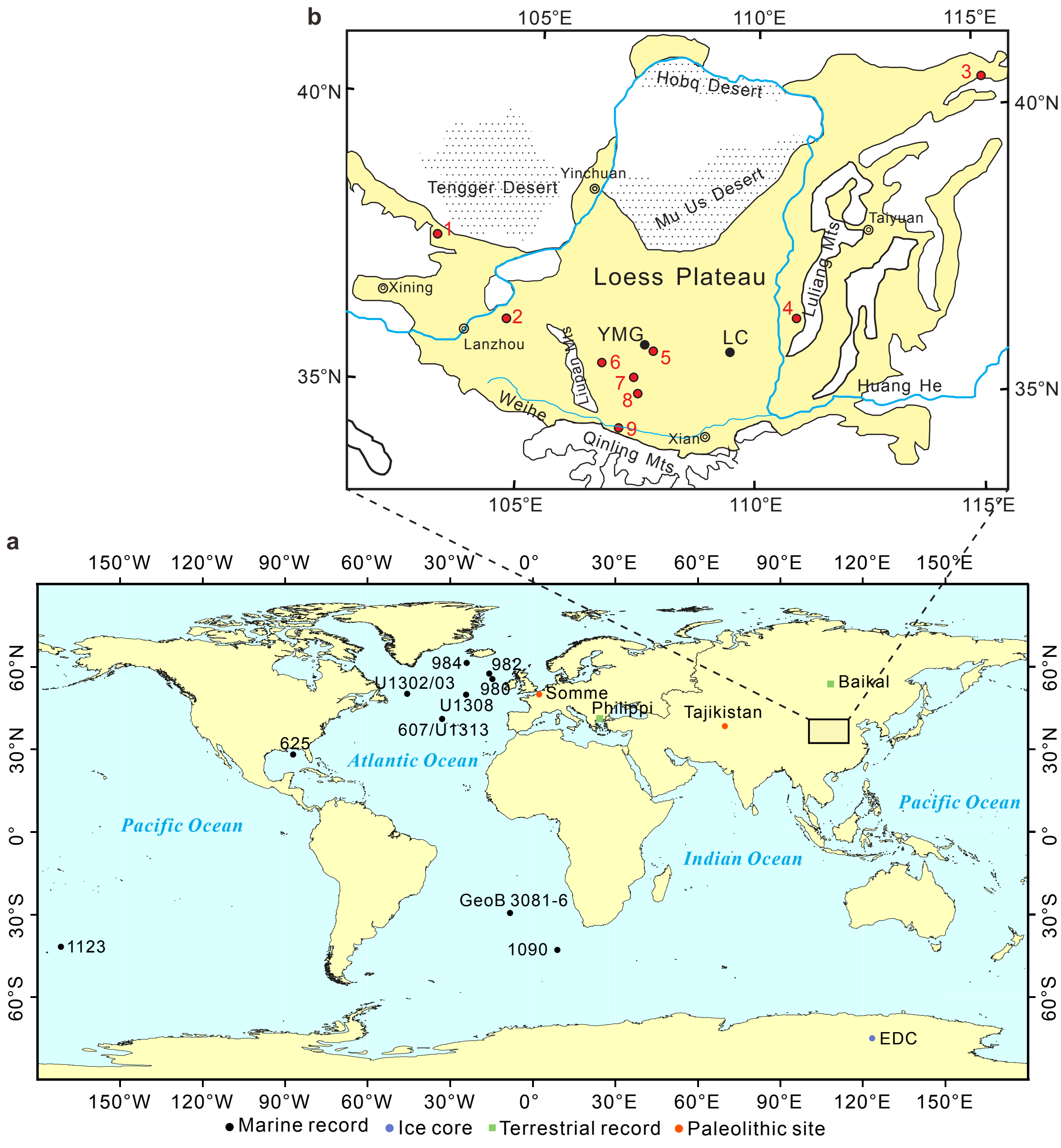

Supplementary Figure 1. Location of paleoclimate and Paleolithic records mentioned in this study. **a**, World map. **b**, Enlarged map of the Chines Loess Plateau. In b, sites 1-9 are the sections shown in Fig. S2. Site 1 refers to the Shagou section[1]; 2 to the Jingyuan section[2]; 3 to the Fanshan section[3]; 4 to the Puxian section[4]; 5 to the Zhaojiachuan section[5]; 6–9 to the Pingliang, Jingchuan, Lingtai and Baoji sections, respectively[4]. **a** was created using ArcGIS10.3 and **b**, modified from Fig. S1 (ref. 6).

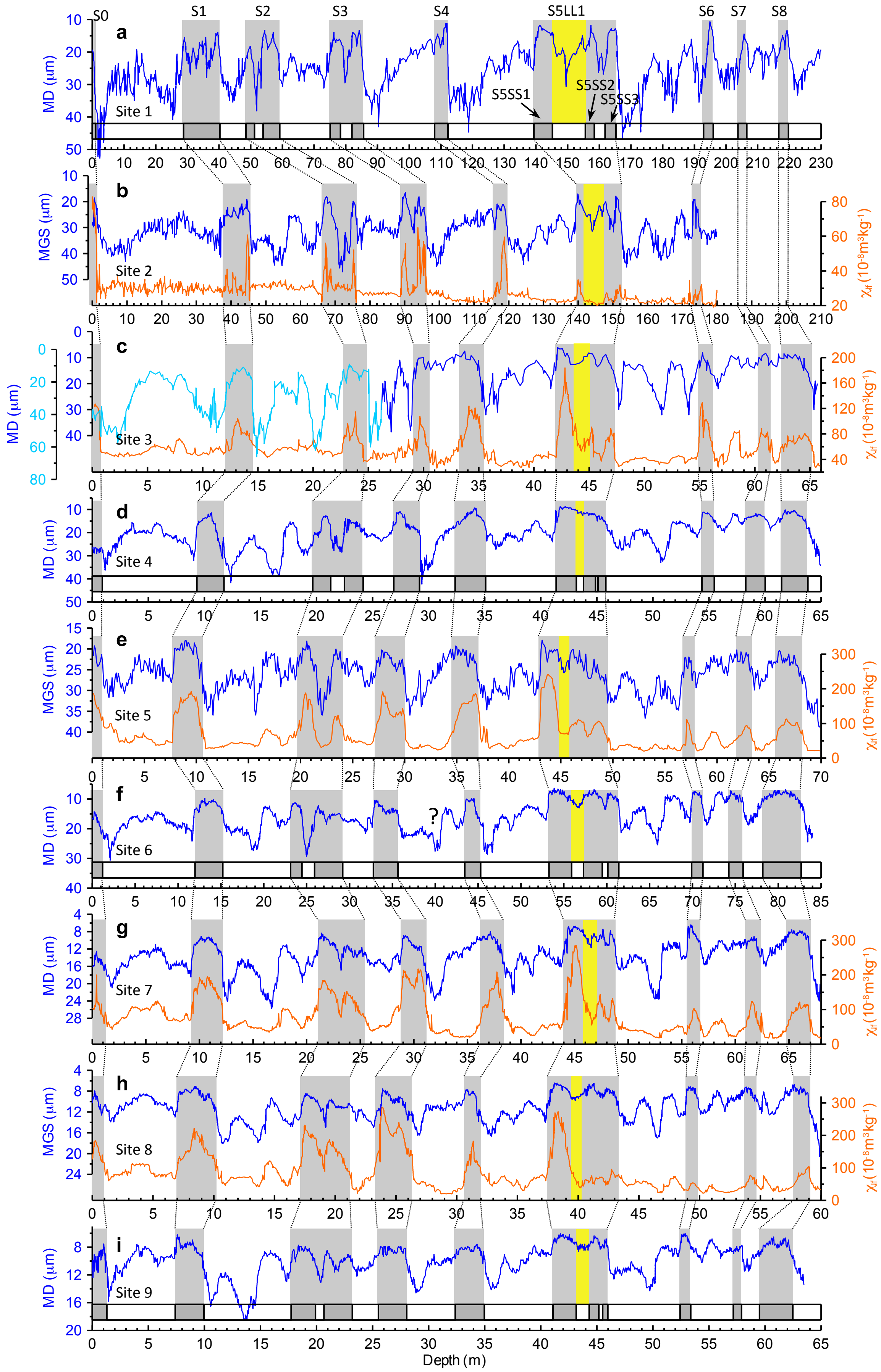

Supplementary Figure 2. Correlation of grain-size records from the loess-paleosol sequences throughout the Chinese Loess Plateau. All of the published magnetic susceptibility (χ_{lf}) records are also shown for comparison. MD and MGS denote median grain-size and mean grain-size, respectively. The lithology column is shown for the sections without χ_{lf} records, with the grey column referring to paleosols and open ones to the loess layers. The span of the paleosols is defined by stratigraphic observation and magnetic susceptibility. The yellow vertical shading highlights the sub loess layer S5LL1 of MIS 14. See Fig. S1 for the section locations and data sources.

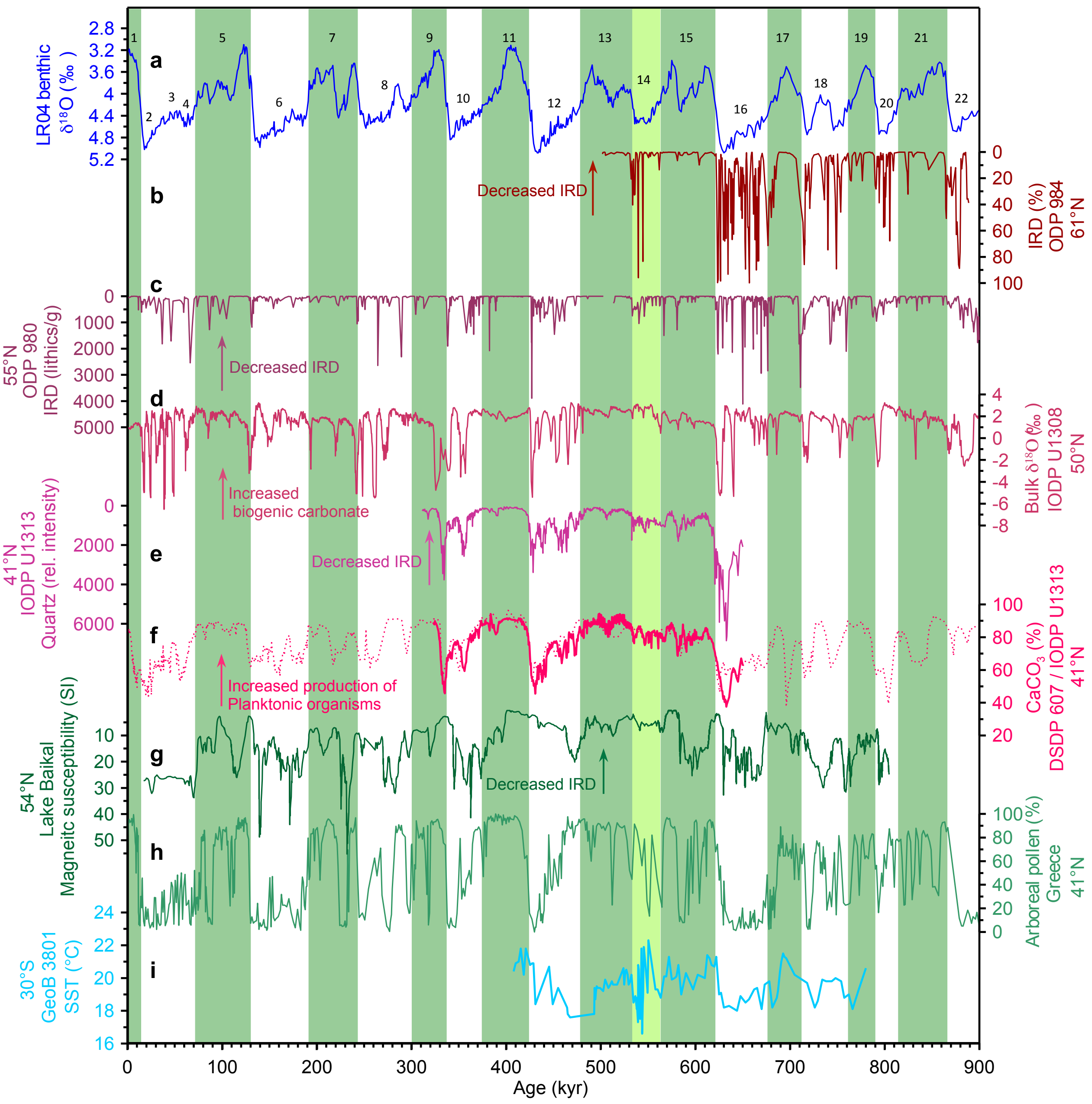

Supplementary Figure 3. Correlation of records from Atlantic, Eurasia continent with benthic δ^{18}O stack LR04. **a**, Benthic stack δ^{18}O[19] . MIS numbering is indicated. **b**, Percentage of ice-rafted debris (IRD) at ODP 984[7]. **c**, IRD number at ODP 980[7,8]. **d**, Bulk δ^{18}O record at IODP U1308[10]. **e**, Relative XRD intensity of quartz at IODP U1313 (a re-occupation of ODP 607)[11]. Quartz content reflects the ice-rafting intensity. **f**, Carbonate percentage at ODP 607 / IODP U1313[11,13]. **g**, The magnetic susceptibility record of Lake Baikal revealing particularly low debris input, which reflects lack of IRD sediments produced by mountain glacier during MIS 14[14]. **h**, Percentage of arboreal pollen in lake deposits from the Philippi Basin in NE Greece[15]. **i**, Sea surface temperature (SST) record from Mg/Ca of core GeoB U3801-6 in low-latitude of South Atlantic[18]. The green shading indicates the interglacial stage defined by the LR04 record[19], and the lighter green one indicates the MIS 14.
